# Recovery of Pasteurization-Resistant *Vibrio parahaemolyticus* from Seafoods Using a Modified, Two-Step Enrichment

**DOI:** 10.3390/foods11050764

**Published:** 2022-03-07

**Authors:** Guadalupe Meza, Hussain Majrshi, Hung King Tiong

**Affiliations:** 1 Department of Biological and Environmental Sciences, University of West Alabama, Livingston, AL 35470, USA; guadalupemaria13@gmail.com (G.M.); h.m.a.601@hotmail.com (H.M.); 2 Clinical Laboratory, Huntsville Hospital, Huntsville, AL 35801, USA

**Keywords:** foodborne pathogen, safety, heat-resistant, pasteurization, seafood, spore, viable-but-non-culturable (VBNC), vibriosis, *Vibrio parahaemolyticus*

## Abstract

Persistent *Vibrio-parahaemolyticus*-associated vibriosis cases, attributed, in part, to the inefficient techniques for detecting viable-but-non-culturable (VBNC) *Vibrio* pathogens and the ingestion of undercooked seafood, is the leading cause of bacterial seafood-borne outbreaks, hospitalizations, and deaths in the United States. The effect of extreme heat processing on *Vibrio* biology and its potential food safety implication has been underexplored. In the present work, environmental samples from the wet market, lagoon, and estuarine environments were analyzed for *V. parahaemolyticus* recovery using a modified, temperature-dependent, two-step enrichment method followed by culture-based isolation, phenotype, and genotype characterizations. The work recovered novel strains (30% of 12 isolates) of *V. parahaemolyticus* from prolonged-heat-processing conditions (80 °C, 20 min), as confirmed by 16S rDNA bacterial identification. Select strains, VHT1 and VHT2, were determined to be hemolysis- and urease-positive pathogens. PCR analyses of chromosomal DNA implicated the *tdh*-independent, *tlh*-associated hemolysis in these strains. Both strains exhibited significant, diverse antibiotic profiles (*p* < 0.05). Turbidimetric and viable count assays revealed the pasteurization-resistant *V. parahaemolyticus* VHT1/VHT2 (62 °C, 8 h). These findings disclose the efficiency of *Vibrio* extremist recovery by the modified, two-step enrichment technique and improve knowledge of *Vibrio* biology essential to food safety reformation.

## 1. Introduction

*Vibrio parahaemolyticus* is a non-spore-forming [1], halophilic [2], and thermophilic [3] Gram-negative bacterium [4]. Attributed to this viable-but-nonculturable (VBNC) [5], infectious agent [3,4] and, hence, uneasy to detect, the number of *V. parahaemolyticus*-associated seafood-borne vibriosis [6,7] is increasing worldwide [3,8]. This gastroenteritis-causing pathogen [9] accounts for ca 45% [6,10] and 0.1% [11] of the total vibriosis and toll of foodborne illnesses (48 million) each year in the US, respectively, and is the leading cause of foodborne infections in China [12]. Infected patients, including all age groups, may carry various symptoms such as watery diarrhea, abdominal cramping, gastroenteritis, nausea, vomiting, fever, wound/soft tissue infections, bacteremia, low blood pressure, blistering skin lesions, and possibly death [6,13]. As such, the US Centers for Disease Control and Prevention (CDC) Cholera and Other *Vibrio* Illness Surveillance (COVIS) [10] and the Foodborne Disease Active Surveillance Network (FoodNet) [14] systems have been closely monitoring this pathogen since 1996. Additionally, the USDA Safe Minimum Internal Temperature chart [15] published cooking conditions for seafood, including shellfish, that requires a minimum internal cooking temperature of 63 °C to ensure safe consumption.

Persistent, increasing vibriosis incidents associated with *V. parahaemolyticus* are generally linked to the consumption of undercooked seafood contaminated with the pathogen [16,17] through their ecological niche [2] and improperly sanitized environment [18]. However, increasing *V. parahaemolyticus* prevalence has been detected in processed, ready-to-eat foods, suggesting that this pathogen can survive the harsh conditions of the food processes [9]. Hemolysin-related genes, such as thermostable-direct hemolysin (*tdh*), thermostable-direct-related hemolysin (*trh*), and thermolabile hemolysin (*tlh*) [19,20], are the primary virulence determinants of *V. parahaemolyticus*. In addition, most clinical strains possess the *trh* gene alongside the urease-producing gene (encoded by the *uh* gene) [21].

Unlike the thermal resuscitation technique, which uses extreme heat [22], VBNC *V. parahaemolyticus* resuscitation requires an enrichment-based cultivation technique, such as a one- or two-step enrichment technique, that involves alkaline peptone water (APW) selection alone or combined nutrient nourishment and APW (or drug) selection, respectively, followed by plating on selective agar containing thiosulfate-citrate-bile-salt-sucrose (TCBS) [23,24]. Of all the enrichment techniques documented, several groups noted that the heated saline enrichment (42 °C) technique was conducive to *Vibrio* growth [25,26,27,28,29,30,31]. However, both Delmore and Crisley [32] and Andrew et al. [33] noted that the heat process using temperatures between 49 and 55 °C was detrimental to this pathogen. Hence, these studies suggest that *V. parahaemolyticus* is a dormant-enabled, non-spore former, as spore former resuscitation requires a much higher temperature [22].

In light of the presence of VBNC *Vibrio* species, laboratories have employed antibody-labeled microscopy assays [13,34,35], gene-specific multiplex PCR [13,19,36], and one- and/or two-step enrichment-based cultivation techniques [37,38] for recovering this type of *Vibrio*. These techniques, however, compromise the feasibility of detecting all other 200 serogroups of cholera-causing *Vibrio* [39], as well as contribute to false-negative results as indigenous contaminants populate [40], which suggest that an efficient technique is needed for the accurate validation of food products for total pathogenic *Vibrio* contaminants, including the VBNC strains.

Collectively, these literature reports suggest the need of the present study to re-evaluate the conventional detection technique and cooking recommendation for *V. parahaemolyticus* containment in seafood products. To the best of our knowledge, the present work has not been noted in peer-reviewed literatures.

*V. parahaemolycus*-associated vibriosis that is deemed to be a persistent foodborne issue with the increasing number of cases worldwide [6,7] may be attributed, in part, to the presence of evasive *V. parahaemolyticus* from a conventional detection technique employed today, alterated cooking conditions, and lack of inspection benefits. The present study devised the conventional technique of APW enrichment [41], followed by heating selection [22], defined as a modified, two-step enrichment technique, to resuscitate/recover a group of heat-dependent, VBNC *V. parahaemolyticus* that could evade the conventional detection technique. The efficacy of the modified, two-step enrichment technique for detecting VBNC *V. parahaemolyticus* strains was simultaneously evaluated with the one-step enrichment technique (i.e., uses APW enrichment alone) [41]. The biology of this type of *V. parahaemolyticus*, which distinguishes it from the generally known, non-spore-like type of this pathogen, was characterized for virulence implications, which provides data for more effective validation for seafood safety. In this study, estuarine, lagoon, and wet-market seafoods were examined to isolate a group of VBNC *V. parahaemolyticus* requiring heat-dependent resuscitation and TCBS agar plating. Virulence determination tests in the literature, such as the Kanagawa phenomenon test, virulence genes PCR test, urease test, and antibiotic disk diffusion test, were subsequently applied to strains of *V. parahaemolyticus* and confirmed by 16S rDNA bacterial identification.

## 2. Materials and Methods

Sample collection. A total of eight shellfish samples (Table 1), including environmental samples (3, crab; 2, oyster) and wet market samples (3, shrimp), were collected from the Joe Patti seafood market (wet market, shrimp collection) around Santa Rosa Island (lagoon, oyster collection) and Gulf Island National beach (estuarine environment, crab collection) of Pensacola, Florida in early Fall (19 September 2017). All packaged samples were kept in an icebox (~1 °C) during transportation back to the laboratory, stored at refrigeration temperature (0–2 °C), and processed within a week. Environmental conditions at sample collection sites were determined to be pH 8.5 and temperature 30.3 °C.

**Figure 1:**
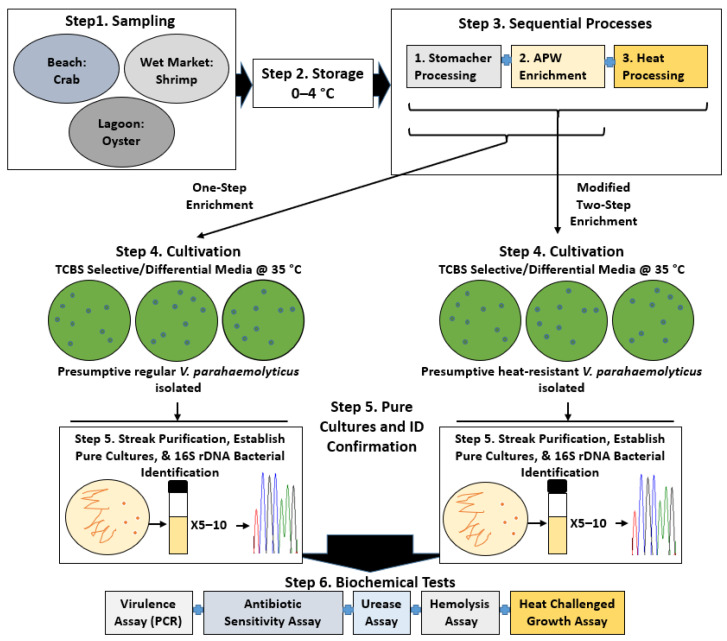
Overview of the *V. parahaemolyticus* study. Step 1, sampling to collect environmental shellfish. Step 2, storage of samples before experimental analyses. Step 3, processes for the recovery of regular and heat-resistant *V. parahaemolyticus*. Step 4, cultivation of presumptive *V. parahaemolyticus* (from processed samples) on selective agar plates containing TCBS. Step 5, streak isolation, culture establishment, and bacterial identification of two forms of *V. parahaemolyticus*. Step 6, biochemical tests to characterize *V. parahaemolyticus* isolates.

Isolation, culture, and storage conditions. Strains of *V. parahaemolyticus* used in this study were isolated in the lab using a two-step enrichment technique described previously [37] with minor modifications (Figure 1). Briefly, microorganisms in a 25 g sample were homogenized in 225 g of enrichment broth containing APW (pH 8.6, 5% *wt/v*) using a Stomacher^®^ 400 Circulator lab blender (Seward, Weber Scientific, Hamilton, NJ, USA), incubated at specific times, and bacterial dilutions were inoculated on thiosulfate-citrate-bile-salt-sucrose (TCBS, Difco, Detroit, MI, USA) agar plates or heat treated (i.e., 80 °C, 20 min followed by 4 °C, overnight) prior to the plating, followed by an incubation of 72 h at 35 °C before visible colonies were streaked on Brain Heart Infusion agar (BHI, Difco, Detroit, MI, USA) containing 3% NaCl for purification, with pure cultures being stored in a sterile solution containing BHI and glycerol (10% *v/v*) at −70 °C. Presumptive *V. parahaemolyticus* isolates were confirmed using 16S rDNA bacterial identification and species-gene-specific PCR (*tlh*). The bacterial cultures were prepared by inoculating thawed cultures (1/100) in sterile fresh culture solution containing BHI (pH 7.4) supplemented with 3% NaCl, followed by overnight incubation (~20 h) at 35 °C and a repeated subculture before experimental analyses, as described previously [18].

Heat challenged viability assay. Sub-cultured, fresh, select strains of *V. parahaemolyticus* were serially diluted to a final cell concentration of 10^2^–10^3^ cfu/mL in LB broth. Dilution aliquots were incubated at 62 °C for 8 h in a water bath, followed by overnight incubation at 35 °C, and were then examined for growth by visible broth turbidity and viable plate count on agar containing BHI and 3% NaCl.

Hemolysis test. Hemolysis activity was examined using sheep erythrocytes [42] or human erythrocytes [16] as described previously with minor modifications. Select strains of *V. parahaemolyticus* sub-cultured twice in sterile BHI broth were spotted (5 µL) onto agar plates (pH 7.4) containing BHI, 1.5% human erythrocytes, and 1% NaCl. Inoculated plates were incubated at 35 °C for 24 h prior to hemolysis validation.

DNA extraction, PCR, and agarose gel electrophoresis conditions. Genomic DNA was prepared with a previously documented bead extraction method [43]. Briefly, pelleted fresh *V. parahaemolyticus* cultures (16 h) were resuspended in sterile DI water and spun down twice before being subjecting to bead collision with sterile micro-size beads (5 µm) in 0.1 mL sterile Tris buffer (10 mM, pH 7.4) to release the cytoplasmic components using a pulsing vortex mixer (SPW Industrial, Laguna Hills, CA, USA). Chromosomal DNA was phased out from cell debris using a high-speed microcentrifuge (VWR, Suwanee, GA, USA) and stored at −20 °C. The quality of DNA was analyzed using a UV spectrophotometer (Thermo Scientific, South San Francisco, CA, USA) and PCR using gene-specific primers.

PCR mixtures for the amplification of genes were formulated according to the GoTaq Flexi DNA Polymerase’s instructions (Promega, Madison, WI, USA). Briefly, each reaction mixture contained 5× PCR buffer (Promega), 0.4 μM of gene-specific primers (Table 2) (IDT, Coralville, IA, USA), 1.5 mM MgCl_2_ (Promega), 0.2 mM deoxynucleoside triphosphate mix (Fisher Scientific, Fair Lawn, NJ, USA), and 1.25 U of GoTaq polymerase (Promega). The PCR conditions were PCR step 1: 1 cycle of 5 min DNA denaturation at 95 °C; step 2: 40 cycles of 1 min DNA denaturation at 95 °C, 40 s primer-dependent annealing, 72 °C of DNA extension (gene-dependent incubation time); and followed by PCR step 3: 1 cycle of extended DNA extension for 10 min at 70 °C before infinite holding at 4 °C in a GeneAmp PCR System 9700 Thermal Cycler (Applied Biosystems, Thermo Scientific).

PCR products (5 µL) were examined in a 1.6% agarose gel (formed in 1× Tris-borate-EDTA buffer) pre-stained with a GelStarTM Nucleic Acid Gel Stain (ratio 5 µL stain: 50 mL gel solution, Lonza Walkersville Inc., Walkersville, MD, USA) using a UV transilluminator.

Urease assay. *V. parahaemolyticus* urease activity was evaluated according to the protocol of a commercial urease testing kit. Briefly, a test reaction containing 1 mL of fresh cell solution of *V. parahaemolyticus* isolates (<20 h) and one tablet of Urease Test Tablets Key Scientific (Hardy diagnostics, Santa Maria, CA, USA) was mixed with a vortex machine and incubated at 37 °C for a maximum of 24 h, or until a pink color indicating a positive test developed.

Antibiotic disc diffusion test. A comparative antibiotic assay for distinguishing strains of *V. parahaemolyticus* was performed using the disc diffusion test with minor modifications [46]. Briefly, BHI agar plates supplemented with 3% NaCl were inoculated for bacterial lawns with 10^8^ cfu/mL fresh *V. parahaemolyticus* cultures (~20 h) by using sterile glass rods. The antibiotic discs (BD; Sparks, MD, USA) chloramphenicol (CHL, 30 µg/disc), ciprofloxacin (CIP, 5 µg/disc), erythromycin (ERY, 15 µg/disc), gentamycin (GEN, 10 µg/disc), nalidixic acid (NAL, 30 µg/disc), neomycin (NEO, 30 µg/disc), streptomycin (STR, 10 µg/disc), and tetracycline (TET, 30 µg/disc) were distantly placed on the lawns. The inoculated plates were incubated for 24 h before antibiotic susceptibility diameters (mm) were determined and then scored per the Clinical and Laboratory Standards Institute (CLSI) M45 [47] and M100-S21(M2) (i.e., found in the antibiotic product user manual) guidelines for *Vibrio* species and enterococci/*Escherichia coli*, respectively.

## 3. Results

### 3.1. Colony Phenotype/Prevalence of V. parahaemolyticus from Thermally Treated Samples

In the present study, VBNC strains of *V. parahaemolyticus* were generated from wet-market and environmental shellfish seafoods. Using a conventional, one-step enrichment (i.e., APW enrichment) and a modified, two-step enrichment technique (i.e., APW enrichment + heat treatment), 12 of 50 isolates were confirmed as two biologically distinct groups, herein named regular and heat-resistant *V. parahaemolyticus*, respectively, and exclusively recovered from oyster samples at all incubation times tested (for the one-step enrichment technique) (Table 3) and 8/48 incubation hours tested (for the two-step enrichment technique) (Table 4 and Table 5). Regular *V. parahaemolyticus* exhibited a 1.4-fold higher number of isolates than the heat-resistant *V. parahaemolyticus*.

### 3.2. Heat Resistance in Heat-Resistant V. parahaemolyticus Vegetative Cells

**Figure 2:**
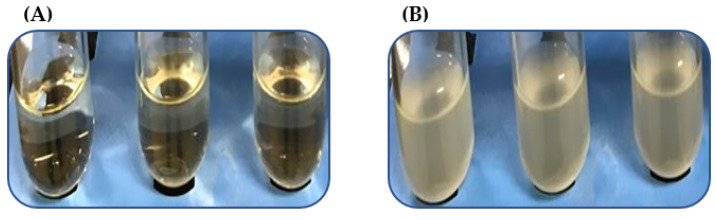
Growth turbidity at post 35 °C incubation of *V. parahaemolyticus* pre-heated at 62 °C for 8 h. *V. parahaemolyticus* VHT1 (**A**) and VHT2 (**B**) were tested in triplicates.

**Figure 3:**
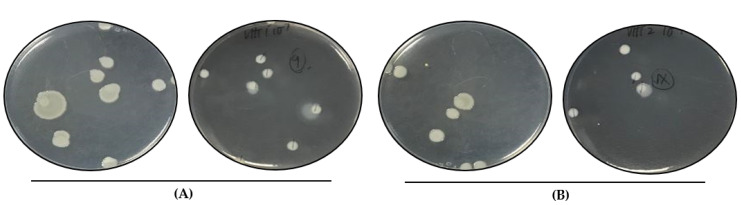
Colony morphology of heated *V. parahaemolyticus* cells. Viable VHT1 (**A**) and VHT2 (**B**) cells heated at 62 °C for 8 h were recovered by plating and incubation at 35 °C. VHT79 (**A**), VHT80, and VHT81 (**B**) derived from the VHT1 and VHT2 colonies were established for identity confirmation analysis. (**A**) left, lid up; right, lid down. (**B**) left, lid up; right, lid down.

We analyzed a subset of heat-resistant strains, VHT1 and VHT2 (Table 5) of *V. parahaemolyticus*, for their susceptibility to the USDA-recommended pasteurization temperature of 62–65 °C for seafood [15]. VHT2 exhibited greater heat tolerance than VHT1, as demonstrated in growth turbidity replications (35 °C) (Figure 2 and Table 6) at post-heat treatment (8 h at 62 °C). It is worth noting that there was no noticeable growth turbidity for both VHT1 and VHT2 through the entire 8 h of incubation at 62 °C (data not shown). The viability of cells with turbidity was confirmed by plating (Figure 3).

### 3.3. Virulence Determinants of the Heat-Resistant V. parahaemolyticus VHT1 and VHT2

- Kanagawa phenomenon

**Figure 4:**
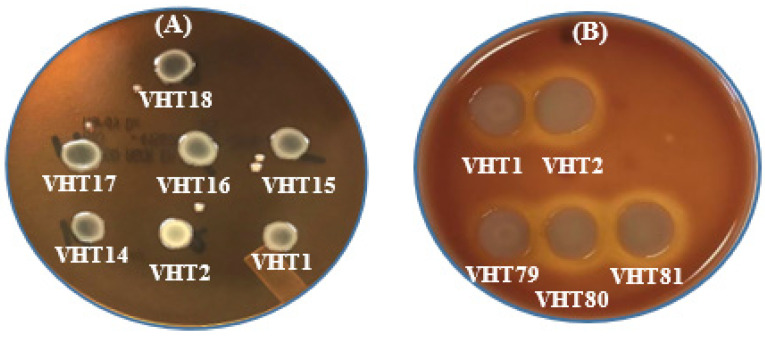
In-vitro hemolysis assay for determining the KP of *V. parahaemolyticus* using sheep erythrocytes (**A**) or human erythrocytes (**B**). VHT1, VHT2, VHT14, VHT15, and VHT16 were heat-resistant strains of *V. parahaemolyticus*; VHT17 and VHT18 were regular strains of *V. parahaemolyticus*; VHT79, VHT80, and VHT81 were derived from VHT1/VHT2 at the post-pasteurization process. The same hemolytic results were observed in at least two separate replications.

The heat resistant (VHT1, VHT2, VHT15, VHT16, VHT79, VHT80, or VHT81) and regular (VHT17 or VHT18) strains of *V. parahaemolyticus* (Figure 4) produced in this study were analyzed for virulence phenotype characterizations, such as the Kanagawa phenomenon (KP) and urease activity. Heat-resistant and regular strains (VHT1, VHT2, VHT15, VHT16, VHT17, and VHT18) examined with sheep erythrocytes did not exhibit positive KP activity as opposed to the use of human erythrocytes (tested in VHT1, VHT2, VHT79, VHT80, and VHT81) (Figure 4 and Table 7).

- PCR amplification of hemolysin genes

**Figure 5:**
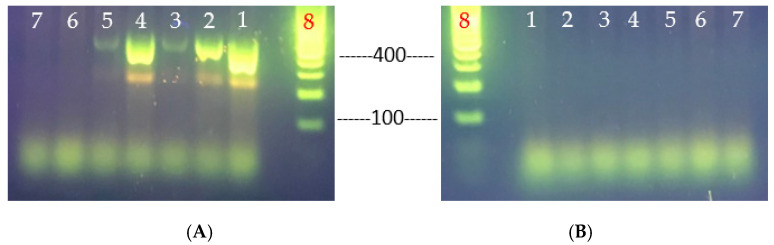
Primer-specific PCR analyses for the determination of *V. parahaemolyticus* virulence genes, *tlh* (**A**) and *tdh* (**B**). *V. parahaemolyticus* strains tested: lane 1, VHT1; lane 2, VHT2; lane 3, VHT14; lane 4, VHT15; lane 5, VHT16; lane 6, VHT17; lane 7, VHT18; and lane 8, 100 bp DNA ladder.

**Figure 6:**
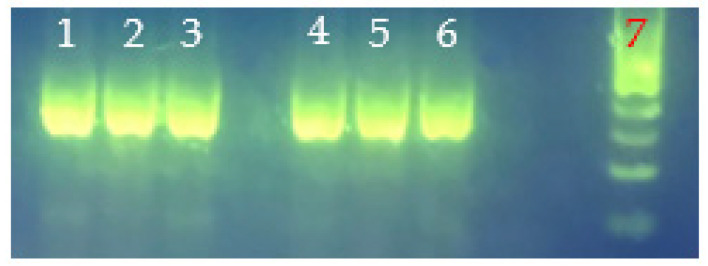
*tlh* amplicons from genomic DNA of *V. parahaemolyticus* strains VHT79, VHT80, and VHT81 for strain purity validation. Lane 1, VHT79; lane 2, VHT80; lane 3, VHT81; lane 4, VHT79 (rep2); lane 5, VHT80 (rep2); lane 6 (rep2); VHT81; and lane 7, 100 bp DNA ladder.

PCR analysis of hemolysin genes (i.e., *tdh*, *tlh*) in select heat-resistant and regular strains of *V. parahaemolyticus* with the gene-specific primers listed in Table 1 revealed *V. parahaemolyticus* with *tdh^−^*/*tlh^−^* (i.e., regular *V. parahaemolyticus* VHT17 and VHT18) or *tdh^−^*/*tlh*^+^ (i.e., heat-resistant *V. parahaemolyticus* VHT1, VHT2, VHT14, VHT15, and VHT16) genotypes (Figure 5 and Table 7) based on expected amplicon sizes (Table 1). Additionally, the derivatives of VHT1 (VHT79) and VHT2 (VHT80 and VHT81) retained the *tlh* gene of their parental strains as demonstrated in Figure 6.

- Urease activity

Urea hydrolysis conditions were adapted from the chemical supplier Hardy Diagnostics (product cat. # K650) since the urea tablets used for urease examination were less laborious to perform than a urea agar-based examination method. Urease activities were detected in all strains with no specific association between intense urease production and each of two forms of *V. parahaemolyticus* (Table 6) detected in this study.

### 3.4. Antibiotic Profile of the V. parahaemolyticus VHT1 and VHT2

**Figure 7:**
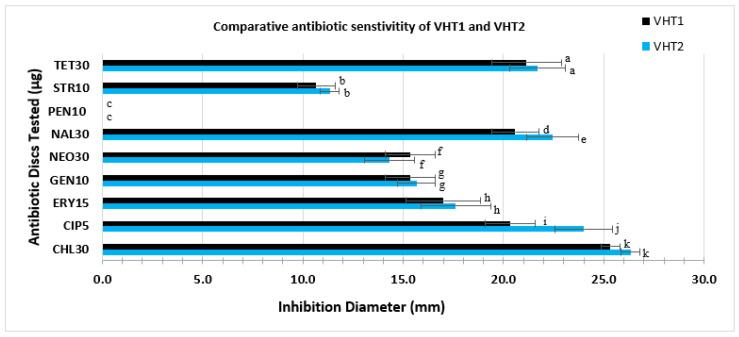
Comparative antibiotic sensitivity profile of VHT1 and VHT2. Antibiotic discs with a specified concentration (µg/mL) used in this study include CHL30, CIP5, GEN10, NEO30, NAL30, PEN10, STR10, and TET30. Data and error bars are the means of at least triplicate replications and the standard deviations from the mean, respectively. Antibiotic activities with a significant difference, set at *p* < 0.05, are given as different letters, whereas antibiotic activities with the same letters are not significantly different.

Antibiotic testing was carried out with a subset of two heat-resistant strains of *V. parahaemolyticus* (i.e., VHT1 and VHT2) obtained in this study. Different antibiotic susceptibility profiles were detected between VHT1 and VHT2. The latter strain was susceptible to more of the antibiotics tested (i.e., susceptible to chloramphenicol, ciprofloxacin, gentamycin, nalidixic acid, and tetracycline) as opposed to the VHT1 strain (i.e., susceptible to chloramphenicol, gentamycin, nalidixic acid, and tetracycline) (Table 8). A significantly (P < 0.05) high level of susceptibility was detected in VHT2 to ciprofloxacin and nalidixic acid as compared with the other strain tested (Figure 7). Both strains were highly resistant to penicillin (Figure 7) and scored an equal MAR index value of 0.22 (Table 8).

## 4. Discussion

The ability of *Vibrio* species to form a viable-but-non-culturable (VBNC) state and become resistant to stresses, such as high salt content, pH, high temperatures [42,48], and antibiotics [49,50] has recently started to shed light on the persistent cases of vibriosis [35,51,52,53,54]. Previous studies separately reported by Mizunoe et al. [5], Okuda et al. [21], and Xu et al. [55] have suggested the presence of genotype and phenotype variabilities of *V. parahaemolyticus,* which could result in detection inconsistency/limit of one or many other detection techniques reported to date.

Conventionally, *Vibrio* spp. or spore-forming bacteria enrichment techniques use nutrient cultivation followed by selective enrichment [23,31,56] or heat treatment followed by cold incubation [22]. In this study, however, we combine SPW enrichment, heat-treatment, and cold incubation for resuscitating *V. parahaemolyticus*. This novel, combined enrichment and heat-selection technique (i.e., modified, two-step enrichment technique) resuscitated a group of environmental, heat-resistant *V. parahaemolyticus* strains (41.7%), in addition to regular *V. parahaemolyticus* strains (58.3%). This suggests the presence of a distinctive genotype and phenotype of *V. parahaemolyticus* that could remain unculturable by many conventional detection/cultivation techniques. Odeyemi noted that *V. parahaemolyticus* is more prevalent in oysters than other kinds of seafood investigated by various groups between 2003 and 2015, as demonstrated in a meta-analysis study [3]. This phenomenon was consistent with the prevalence findings (in this study), whereby *V. parahaemolyticus* could only be retrieved from oysters when analyzed along with other seafood samples. Hence, the observations suggest that oyster may be a *V. parahaemolyticus*’s preferred symbiont, including the heat-resistant strains reported for the first time in this study, compared with other kinds of shellfish seafood which are deemed frequent targets of contamination by *V. parahaemolyticus*. It is worth noting that this technique has not been documented in prior reports, and that the use of an extended SPW enrichment time (i.e., 72 h) in this study is novel (Table 3).

Delmore and Crisley [32] and Andrews et al. [33] noted that *V. parahaemolyticus* is susceptible to temperatures between 49 and 55 °C. Cebrián et al. [57] and Terano et al. [22] noted numerous non-*Vibrio* species of Gram-negative bacteria that are capable of withstanding pasteurization temperature (i.e., 65 °C). The heat-resistant strains of *V. parahaemolyticus* investigated in this study were able to survive extended pasteurization conditions (i.e., 62 °C, 8 h) as opposed to other vegetative cells of spore-forming bacteria [58] or non-spore forming bacteria [57]. This could suggest the differential expression of heat-shock proteins, such as DnaI [42], DnaK [48], GroEL, and GroeS [42,48] chaperones, whose role is implicated in bacterial maintenance of heat-deactivated proteins to revert their structure(s) and function(s) [59]. Both *V. parahaemolyticus* VHT1 and VHT2 strains exhibited different heat-tolerance phenotypes (VHT79, VHT80, VHT81) at post-pasteurization temperature (62 °C), as demonstrated by turbidimetric data (35 °C) (Figure 2) and followed by viable plate count (>8 log cfu/mL cells) data (Figure 3); nevertheless, these strains are susceptible to the resuscitation temperature (80 °C) used to recover them (Table 6). It is worth noting that the heat-resistant colonies of VHT80 and VHT81 derived from VHT2 (i.e., pasteurized VHT2) were different phenotypically and that our findings suggest, for the first time, that *V. parahaemolyticus* could tolerate pasteurization temperatures (62–65 °C). The latter strain (VHT81) appeared to have sticky colonies. This could be attributed to the heat-dependent alteration of Gram-negative bacteria’s outer membrane, which leads to non-lethal LPS damage [60] or homeoviscous adaptation [61]. In contrast to Delmore and Crisley [32], both strains survived a much higher incubation temperature than elevated temperatures between 49 and 55 °C.

KP was prepared using sheep erythrocytes as described by Ming et al. [62], as it is more economical than human erythrocytes. However, agar containing BHI was used for the reason that Wagatsuma agar [16] is costly, and that the *V. parahaemolyticus* strains did not grow well on agar plates containing Luria Bertani. In our study, human erythrocytes were able to demonstrate detectable hemolysis activity, disagreeing with Ming et al. [62]. The findings suggest that the *V. parahaemolyticus* heat-resistant strains VHT1 and VHT2 could possess one or more major hemolysin-producing gene(s) of the virulence strains, such as thermostable direct hemolysin (*tdh*) and/or *tdh*-related hemolysin (*trh*) [38], and that their derivative strains VHT79 (from VHT1), VHT80 (from VHT2), and VHT81 (VHT2) retained their hemolysis phenotypes post pasteurization (Figure 4 and Table 7). It is worth mentioning that all KP^+^ strains tested, including VHT1 and VHT2, did not exhibit hemolysis activity when cultured on agar media supplemented with sheep erythrocytes instead of human erythrocytes (Figure 4 and Table 7).

Wang et al. [63] noted that the Kanagawa hemolytic test could render false virulence results for *V. parahaemolyticus* and that *tdh*-specific PCR analysis could be used to confirm their virulence property. Letchumanan et al. [38] further noted that the non-virulence factor-associated hemolysin gene thermolabile hemolysin (*tlh*) could be employed for detecting total *V. parahaemolyticus*. Our PCR data revealed that regular *V. parahaemolyticus* strains and heat-resistant *V. parahaemolyticus* strains possess *tdh*^−^/*tlh*^−^ and *tdh*^−^/*tlh*^+^ hemolysin genotypes, respectively (Table 7). It is worth noting that this is the first case of environmental *V. parahaemolyticus* with *tlh*^−^. Additionally, the data demonstrated that these KP^+^ *V. parahaemolyticus* strains (i.e., VHT79, VHT80, VHT81) possess *tdh*^−^/*tlh*^+^ virulence factors (Table 7) and that this hemolysin gene is phenotypically (Figure 4) and genotypically (Figure 6) stable to pasteurization conditions.

Xu et al. [55] and others [64,65,66,67,68] noted the presence of urease activity in *V. parahaemolyticus* and that it positively correlates/co-exists with the presence of the thermostable direct hemolysin-related hemolysin (*trh*) gene [21,64,65,68,69,70]. However, the other groups reported that urease activity is usually found in non-clinical strains of *V. parahaemolyticus* [65,66,67,68], which is in opposition to Xu et al. [55]. When urease activity was analyzed for regular (VHT17, 18) and heat-resistant (VHT1, 2, 14, 15, 16) strains of environmentally isolated *V. parahaemolyticus* in this study, both groups exhibited positive urease reaction (Table 7).

Park et al. [65] also noted that the urease and *trh* gene cluster does not positively correlate with the Kanagawa phenomenon (KP). However, the present study revealed that select strains, the heat-resistant, urease^+^ *V. parahaemolyticus* VHT1 and VHT2, were KP^+^ (Table 7). Letchumanan et al. [38] and Chung et al. [71] noted other *V. parahaemolyticus* hemolysins, such as the thermolabile hemolysin (*tlh*) and thermostable direct hemolysin (*tdh*), respectively, that could contribute to KP^+^. Hemolysin gene-specific PCR analyses revealed that the *V. parahaemolyticus* strains VHT1 and VHT2 were *tdh*^−^ but *tlh*^+^ (Table 7). It is worth noting that this is the first report of the discovery of urease^+^, KP^+^ environmental *V. parahaemolyticus* [65].

The different antibiotics used in this study were adapted in part from previous documented work [18,46] and the availability of the antibiotics in the lab. Elexson et al. [72] and Letchumanan et al. [18] noted penicillin/ampicillin-resistant strains of *V. parahaemolyticus* from environmental and cultured seafood, respectively. When the antibiotic susceptibility test was compared between the heat-resistant *V. parahaemolyticus* VHT1 and VHT2, both demonstrated high levels of resistance to penicillin.

The availability of heat-resistant *V. parahaemolyticus* in seafoods suggest the potential cause of vibriosis food outbreak by a heat-resistant group of *V. parahaemolyticus* due to their ability to evade the conventional protocol of cooking and testing [15] and become a food safety threat and vibriosis cause. Our findings suggest, for the first time, that *V. parahaemolyticus* could survive food processing and cooking criteria [15], which could be an overlooked root cause of the persistent vibriosis in the US, and that they could be activated/recovered using the modified, two-step enrichment of combined nourishment (APW, 48 h), heating selection (80 °C, 20 min), and cooling steps. As previously noted, the prevalence of the *V. parahaemolyticus* variant [5,21,55], such as heat-resistant strains of environmental *V. parahaemolyticus*, could be attributed to specific gene transfers within the bacterial complex/mixture community, that could consist of *Vibrio* species and heat-resistant symbionts [73], and induced by global warming [74]. The results revealing positive KP activity and *tlh* amplicons suggest the presence of environmental *V. parahaemolyticus* pathogens [19,20], and that these strains (i.e., VHT1 and VHT2 derived strains, VHT79, VHT80, and VHT81) (Table 5 and Table 7) possess heat-stable hemolysin gene(s), such as *tlh*, and that it encodes a heat-stress protecting product, possibly suggesting a positive correlation between *V. parahaemolyticus* heat resistance (i.e., 62 °C, 8 h) and persistent vibriosis cases, as noted by Ueda et al. [75], whose work determined a positive correlation between thermolabile protein expression and thermotolerance in a member of Gram-negative bacteria. The PCR results confirm that this pathogen could use *tdh*-independent hemolysin(s), such as *trh* [38,71], *tlh* [2,38], VP3048 putative hemolysin [48], and/or others [71] for exhibiting KP activity (Table 7), and that urease-positive *V. parahaemolyticus* strains do not simultaneously possess a *tdh*-encoding gene, as noted by Wang et al. [63]. Additionally, the data suggest that *tdh* and *tlh* hemolysins are not essential in environmental *V. parahaemolyticus* metabolism, as these genes were absent in a subset (*tlh* gene) or all (*tdh* gene) of our culture isolates (Table 6). Further work (i.e., transcriptomic analyses) exploring the transcriptomic response of this group of *V. parahaemolyticus* strains to extreme temperature (i.e., 62 °C) is worthwhile for fostering a seafood processing/cooking and safety regimen. The results demonstrating positive urease activity suggest that both urease-positive heat-resistant and regular *V. parahaemolyticus* (Table 6) could possess a *trh* gene. Several groups reported that bacteria could produce urease when exposed to nitrogen limitation [76], acidic pH [77], or urea availability [78,79]. Berutti et al. [79] noted the increasing level of nitrogen content in environmental water since 2008, which suggests the adoption of urease in environmental *V. parahaemolyticus*. Thus, this indicates that environmental *V. parahaemolyticus* could readily produce urease in response to the urea availability supplemented in the urease assay. The results demonstrating high levels of resistance to penicillin could be attributed to the outer membrane of the Gram-negative bacteria, which interferes with antibiotic drugs’ entry and antimicrobial efficacy [46,80], as well as to the overuse of antibiotics in aquaculture that leads to bacterial resistance [81,82]. VHT2 was relatively more susceptible to ciprofloxacin and nalidixic acid (Figure 7) than VHT1, suggesting an antibiotic-resistance diversity of heat-resistant, environmental *V. parahaemolyticus*. Collectively, the results suggest that the *V. parahaemolyticus* strains of VHT1 and VHT2 (heat-resistant strains) possess *tlh*, *trh* (presumptive), urease, and KP activities.

## 5. Conclusions

A viable-but-nonculturable physiological state is a well-embraced contributing factor to bacterial resistance to, and evasive abilities from, hurdle technology that result in persistent foodborne bacterial infections, including *V. parahaemolyticus*-associated vibriosis. The present study reveals the first recovery of heat-resistant variants of environmental *V. parahaemolyticus* using the modified, two-step enrichment technique of SPW enrichment (step 1), heat enrichment, and refrigeration (step 2). These novel strains possessed *tdh*^−^, Trh^+^ (presumptive), urease^+^, Kanagawa phenomenon, and spore-like characteristics as opposed to the urease^−^/urease^+^, non-spore-like environmental strains determined in previous studies [32,33]. In addition, we disclose, for the first time, *tlh*^−^ *V. parahaemolyticus* [38], urease^+^/KP^+^ environmental *V. parahaemolyticus* [65], and KP analysis inconsistency with sheep blood. Knowledge of different enrichment conditions that are capable of improving *V. parahaemolyticus* recovery, namely, the regular and heat-resistant forms, and understanding the biology of VBNC bacteria, may help detect total *V. parahaemolyticus* in seafoods and improve the accuracy of test results by manipulating the currently employed FDA-recommended conditions [24,30] for *V. parahaemolyticus* detection, both biologically and physically. These findings imply that the detection conditions that are recommended by the FDA may fail with the resuscitation of heat-dependent *V. parahaemolyticus* and that the modified, two-step enrichment technique that was examined should be adopted by *Vibrio* testing labs. In addition, this technique should be further investigated for the potential recovery of other species of VBNC *Vibrio* pathogens of public concern and additional samples from multiple locations are warranted due to these initial/novel results.

## Figures and Tables

**Table 1:** List of environmental seafood samples used in this study.

Seafood Species	Source	Sample #	Storage Condition
Crab	Beach	2	4 °C
Shrimp	Wet-market	3	4 °C
Oyster	Lagoon	3	4 °C

**Table 2:** Primers used in this study.

Gene ID	Primer	Amplicon Size	Melting Temperature	Annealing Temperature	Source
16S-515	F-GTGCCAGCMGCCGCGGTAA	900	65.2	56	[44]
16S-1391	R-GACGGGCGGTGTGTRCA		59.8	56	
*tdh*	F-GTARAGGTCTCTGACTTTTGGAC	229	66	56	[45]
	R-CTACAGAATYATAGGAATGTTGAAG		66	56	
*tlh*	F-AAAGCGGATTATGCAGAAGCACTG	450	70	56	[45]
	R-GCTACTTTCTAGCATTTTCTCTGC		68	56	

**Table 3:** Colony-forming profiles of presumptive *V. parahaemolyticus* on TCBS agar media following the one-step enrichment method. Samples were plated after specific incubation times on TCBS. Bacterial colonies were identified with 16S rDNA sequencing.

Seafood Species	Enrichment Only Incubation (h)						
	0	4	8	12	24	48	72
Crab	−	−	−	−	−	−	−
Shrimp	+	+	+	+	−	−	−
Oyster	+	+	+	+	+	+	+

−, no V. parahaemolyticus detected; +, V. parahaemolyticus detected.

**Table 4:** Colony-forming profiles of presumptive *V. parahaemolyticus* on TCBS agar media following the two-step enrichment method. Enriched samples (0–72 h) were heated for 20 min at 80 °C and plated on TCBS agar. Bacterial colonies were identified with 16S rDNA sequencing.

Seafood Species	Enrichment Incubation (h) + Heat ^1^						
	0	4	8	12	24	48	72
Crab	−	−	−	−	−	−	−
Shrimp	+	+	+	+	−	−	−
Oyster	−	−	+	−	−	+	−

^1^ Heat processing (80 °C, 20 min) followed after each of the specific nourishment incubation times; −, no *V. parahaemolyticus* detected; +, *V. parahaemolyticus* detected.

**Table 5:** List of *V. parahaemolyticus* isolates identified ^1^ in this study.

Isolate ID ^1^	Isolation Mode ^2^	Enrichment Time (h)	Sample Type	Source
VHT1	Modified two-step	48	Oyster	Lagoon
VHT2	Modified two-step	48	Oyster	Lagoon
VHT14	Modified two-step	48	Oyster	Lagoon
VHT15	Modified two-step	48	Oyster	Lagoon
VHT16	Modified two-step	48	Oyster	Lagoon
VHT17	one-step	24	Oyster	Lagoon
VHT18	one-step	24	Oyster	Lagoon
VHT20	one-step	24	Oyster	Lagoon
VHT21	one-step	24	Oyster	Lagoon
VHT22	one-step	72	Oyster	Lagoon
VHT25	one-step	72	Oyster	Lagoon
VHT26	one-step	72	Oyster	Lagoon
VHT79	Pasteurization	NA	NA	VHT1 derivative
VHT80	Pasteurization	NA	NA	VHT2 derivative
VHT81	Pasteurization	NA	NA	VHT2 derivative

^1^ Bacterial identity was determined by 16S rDNA gene sequencing; VHT79, derived from pasteurized *V. parahaemolyticus* VHT1; VHT80 and VHT81, derived from pasteurized *V. parahaemolyticus* VHT2; ^2^ The enrichment type used in isolating different strains of *V. parahaemolyticus*: modified, two-step, APW enrichment (at specific times) and heating selection; one-step, APW enrichment only; pasteurization at 62 °C (8 h); NA, not applicable.

**Table 6:** Growth of *V. parahaemolyticus* strains VHT1 and VHT2 at post-heat treatment.

Isolate ID ^1^	62 °C ^2^	80 °C ^3^
VHT1	+	−
VHT2	+++	−

^1^ Heated VHT1 and VHT2 were plated, isolated, cultured, and named as VHT79 and VHT80/VHT81, respectively, as demonstrated in the *V. parahaemolyticus* strain list in this study; ^2^ *V. parahaemolyticus* cells in BHI broth were heated for 8 h before overnight incubation at 35 °C; ^3^ *V. parahaemolyticus* cells in BHI broth were heated for 20 min before overnight incubation at 35 °C. Relative to the BHI-broth-only tube control, the viability of the cells were recorded as “+” or “−“ turbidity, indicating cells survived or were killed in the test. Each strain was tested in triplicate replications.

**Table 7:** Virulence phenotype (urease activity) and genotypes (*tdh*/*tlh* genes) of 7 strains of *V. parahaemolyticus*.

Isolate ID	Urease Activity ^1^	*tdh* ^2^	*tlh* ^2^	*KP* ^3^	*KPs* ^4^
VHT1	+++	−	+	+	−
VHT2	++	−	+	+	−
VHT14	++	−	+	ND	−
VHT15	++	−	+	ND	−
VHT16	+	−	+	ND	−
VHT17	+	−	−	ND	−
VHT18	++	−	−	ND	−
VHT79	ND	ND	+	+	ND
VHT80	ND	ND	+	+	ND
VHT81	ND	ND	+	+	ND

^1^ Relative urease activity (+, mild; ++, moderate; +++, high) was measured by comparison with the negative (−) and positive (+) controls; ^2^ Gene availability (+, present; −, absent) was determined by the visibility, as well as the specified size, of the PCR amplicons at post-gel electrophoresis; ^3,4^ Kanagawa phenomenon, contained human erythrocytes (KP) or sheep erythrocytes (KPs), was scored positive (+) for beta, complete hemolysis activity. ND, not determined.

**Table 8:** Antibiotic susceptibility interpretive data for the heat-resistant *V. parahaemolyticus* strains VHT1 and VHT2.

	Susceptibility Interpretive Data *					
	VHT1			VHT2		
Antibiotic	Resistant	Intermediate	Sensitive	Resistant	Intermediate	Sensitive
CHL (30 µg)			v			v
CIP (5 µg)		v				v
ERY (15 µg)		v			v	
GEN (10 µg)			v			v
NAL (30 µg)			v			v
NEO (30 µg)		v			v	
PEN (10 µg)	v			v		
STR (10 µg)	v			v		
TET (30 µg)			v			v
MAR Index	0.22			0.22		

* Adapted from CLSI Document M100-S21 (M2): Disk Diffusion Supplemental Tables, Performance Standards for Antimicrobial Susceptibility Testing. Key: MAR, multiple antibiotic resistance. v, antibiotic susceptibility result.

## Data Availability

Not applicable.
